# Synthesis of Tetragonal BaTiO_3_ Nanoparticles in Methanol

**DOI:** 10.3390/nano15161226

**Published:** 2025-08-12

**Authors:** Nasser Mohamed-Noriega, Julia Grothe, Stefan Kaskel

**Affiliations:** 1 Department of Inorganic Chemistry, Dresden University of Technology, Bergstrasse 66, 01069 Dresden, Germany; julia.grothe@tu-dresden.de (J.G.); stefan.kaskel@tu-dresden.de (S.K.); 2 Faculty of Mechanical and Electrical Engineering, Universidad Autónoma de Nuevo León, Av. Universidad s/n., Cd. Universitaria, San Nicolás de los Garza 66451, Mexico

**Keywords:** barium titanate, tetragonal, methanol, oleic acid, solvothermal, non-hydrolytic

## Abstract

BaTiO_3_ (BT) is an essential material for many applications due to its dielectric, ferroelectric, and piezoelectric properties; nevertheless, it has been reported to possess a “critical size” in the nanoscale below which its outstanding properties are lost and the paraelectric cubic phase is stabilized at room temperature instead of the tetragonal phase. This value depends on multiple factors, mostly resulting from the synthesis route and conditions. Especially, internal stresses are known to promote the loss of tetragonality. Stresses are commonly present in water-containing synthesis routes because of the incorporation of hydroxyl groups into the oxygen sublattice of BaTiO_3_. On the other hand, the use of an organic solvent instead of water as a reaction medium overcomes the mentioned problem. This work presents a one-pot water-free solvothermal treatment of a Ti(O-iPr)_4_-Ba(OH)_2_·8H_2_O sol in methanol in the presence of small amounts of oleic acid, which allows the synthesis of spherical crystalline BT nanoparticles (from ~12 nm to ~30 nm in diameter) at temperatures as low as 100 °C with a cubic/tetragonal crystal structure confirmed by powder XRD, but predominantly tetragonal according to the Raman spectra. The retention of the tetragonal crystal structure is attributed to the lack of lattice hydroxyls (confirmed by FTIR spectroscopy) resulting from the use of an organic solvent (methanol) as reaction medium. To the best of the author’s knowledge, this synthesis approach is the first report of tetragonal BT nanoparticles synthesized in methanol without the addition of extra water and without the need for a post-synthetic calcination step.

## 1. Introduction

Ferroelectric materials such as BaTiO_3_ (BT) serve as essential components in a wide range of electronic applications, including capacitors, ultrasonic transducers, actuators, sensors, energy harvesting devices, ferroelectric memories, non-linear optical elements, and, more recently, promising photovoltaic and electrocaloric technologies [1]. The effectiveness of these applications depends on the presence and activity of the unique properties inherent to ferroelectric materials, which are influenced by various factors, including particle size and morphology, crystallite size, defect characteristics, surface chemistry, and interface properties. Notably, particle size exerts a significant impact on performance. Several studies have identified a “critical size” threshold for ferroelectric materials below which their characteristic properties are diminished or lost; for BT, reported critical sizes range from 100 nm down to 20 nm [2,3,4,5,6,7]. This threshold is marked by a lowering of the ferroelectric-to-paraelectric transition temperature (Curie temperature, Tc) as particle size decreases, eventually falling below room temperature. As a result, the high temperature paraelectric phase becomes stable at room temperature, leading to the loss of ferroelectric properties. Since the initial discovery of this phenomenon by C. Jaccard et al. circa 1950 [8], extensive research has explored the origins of this critical size. Despite considerable advances over recent decades, numerous questions remain, and a single definitive explanation has yet to emerge. Instead, multiple potential causes have been proposed [2,3,4,6,7,9,10,11,12,13,14,15,16]. It is apparent, however, that the critical size is not intrinsic but rather dependent on synthesis and preparation methodologies.

Among various ferroelectric materials, perovskites are the most prevalent, with barium titanate (BT) being particularly prominent. BT was the first oxide ceramic identified to exhibit ferroelectricity (although the phenomenon was initially discovered in Rochelle salt by Valasek in 1920) and remains one of the most extensively studied ferroelectric compounds [17]. BT possesses four stable polymorphs—cubic, tetragonal, orthorhombic, and rhombohedral—arranged from highest to lowest temperature; except for the cubic phase, all are non-centrosymmetric and thus demonstrate ferroelectric properties. In its bulk form, ***B***T is tetragonal at room temperature and transitions to the cubic paraelectric phase above 120 °C (Tc).

Commercially, BT is typically synthesized at elevated temperatures (600–1200 °C), commonly via solid-state reactions or thermal decomposition of appropriate precursors [13,18,19]. These conventional approaches generally yield larger, aggregated particles rather than isolated nanoparticles. Consequently, numerous low-temperature (<300 °C) techniques have been established to facilitate the formation of BT nanoparticles, with hydrothermal and sol–gel methods (both aqueous approaches) being among the most widely adopted [13,18,20,21,22]. However, the literature reports that employing water as a reaction medium—especially under high pressure—favors significant incorporation of protons and hydroxyl ions into the oxygen sublattice of BT nanoparticles, resulting in internal stresses and potentially causing loss of tetragonal symmetry [23,24,25,26,27,28,29,30]. Hennings et al. (1992) [24] further confirmed that hydrothermal conditions in particular enhance this ion incorporation. Moreover, nanoparticles synthesized via these methodologies generally require subsequent calcination at elevated temperatures (400–600 °C) to eliminate organic residues and induce crystallization, including transformation from the paraelectric cubic phase to the ferroelectric tetragonal phase. Unfortunately, such high-temperature treatments promote crystal growth and sintering, thereby diminishing the intrinsic advantages associated with nanoscale materials.

Employing an organic solvent in place of water has been proposed as a solution to the aforementioned challenges, as noted by M. Niederberger [31] and A. Vioux [32], owing to the distinct mechanisms involved in forming M-O-M bonds. In aqueous syntheses, oxygen is sourced from water molecules, whereas in non-aqueous systems, it is supplied by the solvent or the organic component of the reactant. Notably, non-hydrolytic conditions afford improved control over both the hydrolysis and condensation steps during sol–gel processes, and the solvent can serve as a ligand to regulate nanoparticle growth and agglomeration [33]. Consequently, preparing BT nanoparticles using organic solvents—either solvothermally or via non-hydrolytic sol–gel techniques—appears promising. Despite these apparent advantages and the success observed with other oxides [31,32,34], reports on this methodology remain limited. To the best of the author’s knowledge, the earliest report of BT synthesis in a non-aqueous medium (non-solvothermal) dates to 1984, when R. G. Dosch et al. produced thin BT films by coating substrates with a sol derived from reacting titanium alkoxide with barium hydroxide hydrate in methanol, followed by heat treatment in air [35]. The first synthesis of BT powders (non-solvothermal) was reported in 1995 by Takashi Hayashi et al., who generated nanoparticles by reacting titanium (IV) isopropoxide with a methanol suspension of barium hydroxide hydrate, incorporating various amounts of water as a cosolvent [36]. It was not until 2004 that Niederberger et al. synthesized BT powders solvothermally in an organic solvent without additional water, by reacting barium metal with benzyl alcohol and subsequently introducing titanium (IV) isopropoxide [37]. Since then, several studies have investigated non-aqueous synthesis of BT powders [38,39,40,41,42,43,44], classifiable into three categories based on reactant type: alkoxide-hydroxide [35,45,46,47,48], mixed-alkoxide [21,37,49,50,51,52], and TiO_2_-hydroxide (conversion) [53,54,55]. Each category can further be subdivided depending on whether water is used as a cosolvent or if only organic solvents are employed. Among these approaches, alkoxide-hydroxide and mixed-alkoxide methods employing water as a cosolvent are most extensively studied, as water facilitates hydrolysis of alkoxides and dissolution of sparingly soluble barium hydroxide. However, the use of water introduces issues comparable to those faced in aqueous synthesis, such as proton and hydroxyl incorporation, which compromises tetragonal symmetry. As water is not fundamentally required for M-O-M bond formation in non-aqueous systems, a solvent capable of dissolving both alkoxides and barium hydroxide offers a compelling alternative to circumvent these limitations.

To investigate this hypothesis, we evaluate methanol as a reaction medium, given its ability to dissolve all reactants. Herein, we present a single-step, water-free solvothermal method, wherein Ti(O-iPr)_4_ reacts with a Ba(OH)_2_·8H_2_O–methanol solution, with oleic acid (OA) serving as a capping agent, to produce isolated tetragonal BT nanoparticles.

## 2. Materials and Methods

### 2.1. Chemicals and Reagents

Barium hydroxide octahydrate (Ba(OH)_2_·8H_2_O, >98%), titanium (IV) isopropoxide (Ti[OCH(CH_3_)_2_]_4_, 97.0%), and methanol (99.8% anhydrous) were purchased from Sigma-Aldrich (St. Louis, MO, USA). Oleic acid (C_18_H_34_O_2_, 90.0%) was acquired from Alfa Aesar (Haverhill, MA, USA). All chemicals used in this work were used as received.

### 2.2. Synthesis of BT Nanoparticles

In a typical synthesis, 0.676 g (2.14 mmol) of barium hydroxide octahydrate was evacuated and flushed with argon at room temperature prior to the addition of 20 mL anhydrous methanol. The solution was then heated to 50 °C and stirred for ~20 min before the addition of 0.641 mL (2.14 mmol) of titanium (IV) isopropoxide; the solution turned rapidly from slightly cloudy to white, indicating the rapid hydrolysis of the alkoxide. After ~3 min, 0.203 mL (0.643 mmol) of oleic acid (OA) was added (equivalent to an OA/BT molar ratio of 0.3) and stirring was continued for 1 h, a total of four concentrations of OA were investigated (OA/BT of 0, 0.3, 0.6, and 1). The final solution was transferred to an autoclave to perform a solvothermal treatment in a pre-heated oven at different temperatures (80 °C, 100 °C, 150 °C, and 200 °C) and for different lengths (3, 6, 24, and 48 h); all solvothermal reactions were conducted using a stainless steel Berghof DAB-2 Autoclave equipped with a Teflon lining. This apparatus supports a maximum operating temperature of 250 °C and a pressure rating up to 200 bar, thereby preventing methanol volatilization under the reaction conditions utilized. Finally, the powder was collected by centrifugation, washed with a mixture of water and ethanol (1:1), and dried at 80 °C overnight on a Petri dish.

### 2.3. Nanoparticles Characterization

Powder XRD and Raman Spectroscopy were used to determine the crystal structure of the nanoparticles. XRD data were collected with a Panalytical X’Pert diffractometer (PANalytical B.V., Almelo, The Netherlands) in reflection mode at room temperature, using a Cu-Kα_1_ radiation (λ = 0.15405 nm); the samples were measured over the 2θ range from 21° to 55°, with a step size of 0.013°. The Raman spectra were collected with a Renishaw 3000 Raman microscope (Renishaw plc., Gloucestershire, UK) with a 532 nm laser at room temperature; the samples were measured over the range from 100 cm^−1^ to 800 cm^−1^. The size and morphology of the particles were determined by the analysis of SEM images—a Zeiss DSM-982 Gemini equipment was used (Carl Zeiss NTS GmbH, Oberkochen, Germany). The images were taken at an acceleration voltage of 6 kV and a working distance of 5 mm. The samples were prepared by dispersing the dry powders on a glued carbon pad; all samples were sputtered with gold. The image analysis was performed manually with the aid of the software Image-Pro Plus (Version 6.1) by outlining the perimeter of the particles (between 50 and 100 particles) to calculate the mean diameter and the roundness (${Perimeter}^{\wedge }2/4*\pi *Area$). Moreover, the particle size distributions are presented as “Box-Plots”—the Box contains the percentiles between the 25th and the 75th, while the whiskers extend to the 95th and 5th percentile, and the maximum and the minimum are shown as single points. To confirm the surface modification of the particles, FTIR spectroscopic analysis was performed using a Vertex 70 spectrometer (Bruker Scientific Co., Ltd., Billerica, MA, USA); the measurements were performed on the middle infrared spectrum with a “Golden-Gate” accessory (single reflection diamond attenuated total reflection). Finally, thermal analyses (TG–DTA) were carried out to determine the Curie temperature of the nanoparticles and to calculate the amount of organic moieties at the surface of the particles. A Netzsch STA-409 simultaneous TG–DTA equipment (Netzsch GmbH & Co. Holding KG, Selb, Germany) was used; the measurements were performed in air on a corundum crucible in the temperature range from room temperature to 1100 °C at a heating rate of 5 K/min.

## 3. Results and Discussion

### 3.1. OA Concentration

**Figure 1:**
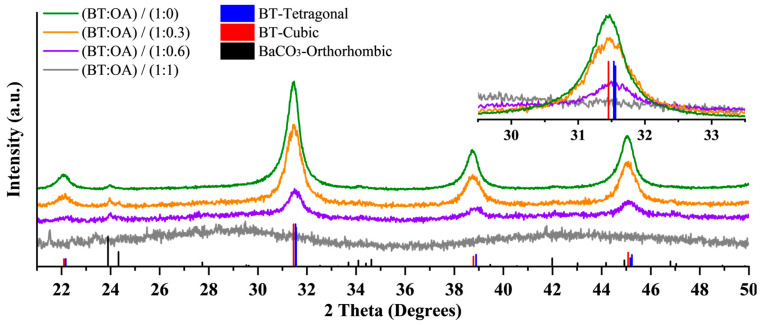
Diffractograms of BT nanoparticles synthesized solvothermally at 200 °C for 3 h at four different OA concentrations. As a reference, the relative peak intensities for the tetragonal (PDF No. 98-005-7485) and cubic (PDF No. 98-008-0552) phases of BT are shown, as well as for BaCO_3_ (PDF No. 01-074-2663). See Figure A2, Figure A3 and Figure A4 for phase deconvolution and Rietveld refinement.

The influence of OA was investigated by synthesizing BT nanoparticles via a solvothermal method at 200 °C for 3 h, varying the OA amount. The molar ratio of OA to theoretical BT yield (mol OA/mol BT) ranged from 0 to 1. All samples, except for those prepared with the highest OA content (1 mol), which was amorphous, exhibited crystalline BT with different degrees of crystallinity (Figure 1); increasing OA concentration corresponded with decreased crystallinity. Notably, the sample synthesized without OA contained significant amounts of BaCO_3_, while those synthesized with OA only displayed traces. According to diffractograms, the three crystalline samples presented a cubic/tetragonal crystal structure; for quantitative analysis of crystal structure and phase composition/fraction, Rietveld refinement was conducted on the diffractograms (Figure A2, Figure A3 and Figure A4), enabling deconvolution of overlapping reflections from cubic and tetragonal phases. As summarized in Table A1, results indicate the sample without OA is predominantly cubic (15.1% tetragonal). The addition of 0.3 mol OA considerably increased the formation of the tetragonal phase (56% tetragonal). At higher OA concentrations (0.6 mol), both the tetragonal fraction decreased (36.9% tetragonal) and the amorphous content increased, aligning with the reduced peak intensity observed.

**Figure 2:**
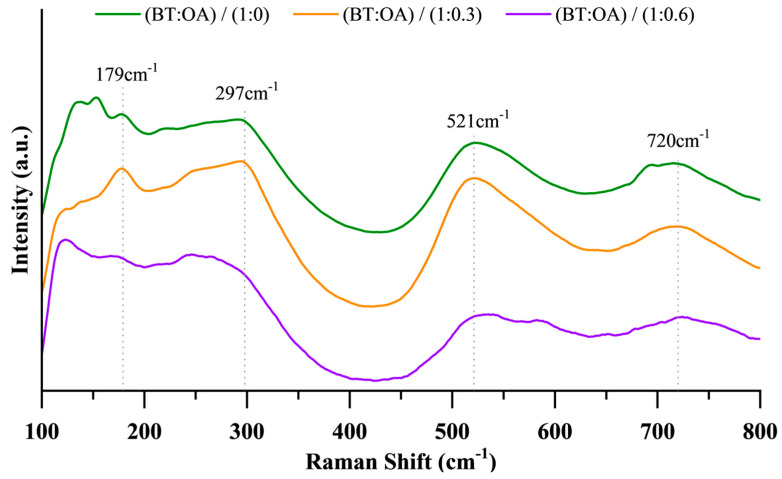
Representative Raman spectra of BT nanoparticles synthesized solvothermally at 200 °C for 3 h at three different OA concentrations. The representative bands for tetragonal BT are shown with dotted lines.

Furthermore, Raman spectroscopy’s sensitivity to symmetry variations serves as an effective method for distinguishing between polymorphs. As depicted in Figure 2, the Raman spectra of all three crystalline samples exhibit the characteristic vibrational modes of BT at ~180 cm^−1^, ~300 cm^−1^, ~520 cm^−1^, and ~720 cm^−1^, corresponding in the literature to the E(TO+LO)/A_1_(TO), E(TO+LO)/B_1_(TO), A_1_(TO), and A_1_(LO)/E(LO) modes, respectively [52,56,57,58,59]. Especially, the mode near 300 cm^−1^ is regarded as indicative of tetragonality. While the Raman data confirms the presence of the tetragonal phase, they do not preclude the possible existence of the cubic phase. Prior studies attribute the simultaneous occurrence of cubic and tetragonal symmetries to the coexistence of distinct phases, likely manifesting as a core–shell structure with a tetragonal core and a cubic shell [4,7,29,60,61,62,63,64].

It is evident that OA influences not only the degree of crystallinity but also the stability of the tetragonal phase. Both high concentrations of OA (0.6 mol) and its absence diminish sample tetragonality, whereas moderate amounts (0.3 mol) enhance it, potentially due to internal stress effects. As outlined in the introduction, the stability of the tetragonal phase on the nanoscale may be affected by several factors favoring the cubic structure. The predominant hypotheses include elastic constraints, structural defects, depolarization phenomena, and the lack of long-range cooperative interactions, as described by M. H. Frey et al. [4]. Therefore, based on current findings and literature, it is reasonable to conclude that excessive OA induces pronounced compressive stress in nanoparticles, promoting the cubic phase, whereas limited OA allows for the dissipation of internal stresses and stabilization of the tetragonal phase. Additionally, Xiaohui Wang et al. and Changqing Jin et al. have demonstrated [9,65] that compressive stress supports the cubic phase. They propose that stress relief may occur via polydomain formation [2,4,66] or crystal symmetry transformation; other mechanisms of stress release may achieve similar effects.

In order to assess the “internal stress” hypothesis, diffractograms were analyzed using the Williamson–Hall (W–H) method. The resulting crystallite size and lattice strain data are presented in Table A2; for comparison, crystallite sizes were also obtained via the Scherrer equation. Since the Rietveld refinement indicated the presence of both tetragonal and cubic phases within the nanoparticles, potentially due to a core–shell structure, then, the W–H analyses were performed on the individual deconvoluted phase components. Application of the W–H method to the tetragonal phase showed an increase in crystallite size from approximately ~5.8 nm for samples without OA to about ~7.0 nm at the highest OA concentration tested (0.6 mol), along with a slight decrease in micro-strain from ~0.0074 to ~0.0055. Similarly, results from the Scherrer equation for specific reflections showed crystallite sizes increasing from about ~8.7 nm to ~9.9 nm as OA content increased. When multiple reflections were averaged, the Scherrer method gave crystallite sizes rising from around ~11.8 nm to ~12.6 nm for the tetragonal samples. For the cubic phase fraction, both the W–H and Scherrer methods showed consistently smaller crystallite size estimates, relative to the tetragonal phase at the corresponding OA concentrations, indicating that stabilization of the cubic phase occurs at reduced coherent domain sizes. This observation corresponds with established tendencies for nanoscale effects and surface-induced compressive stress to promote the higher-symmetry cubic structure in BT. Additionally, the calculated lattice parameter using the (110) reflection remained within the expected range for BT (~0.400 nm) for both phases. A modest increase in the cubic phase lattice parameter compared to the tetragonal phase was observed at each OA concentration, which may reflect a slight lattice expansion associated with residual compressive stress induced by the OA. This behavior is consistent with previous reports where surface ligands stabilize the cubic phase by mitigating tetragonal distortion [67,68].

**Figure 3:**
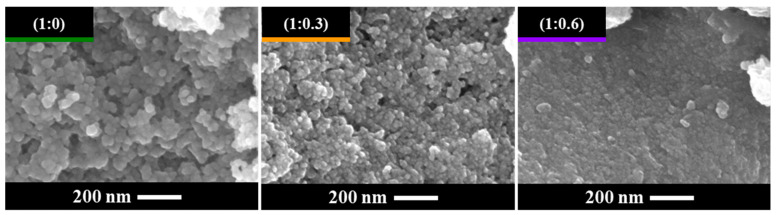
Representative SEM images of BT nanoparticles synthesized solvothermally at 200 °C for 3 h at three different OA concentrations.

**Figure 4:**
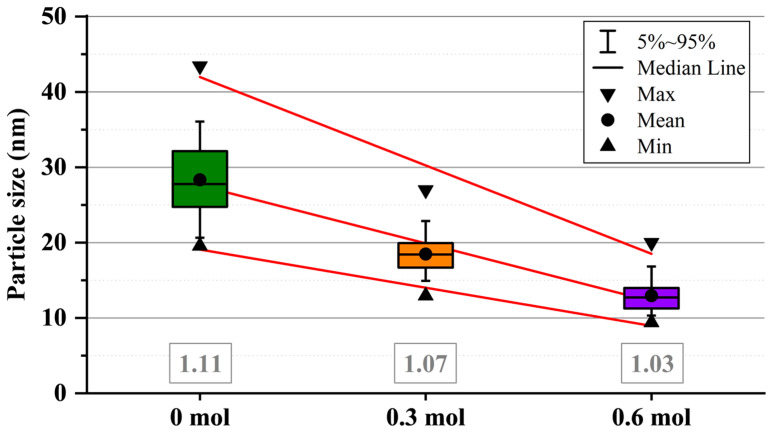
Particle size (from SEM images) of BT nanoparticles synthesized solvothermally at 200 °C for 3 h at three different OA concentrations; the value for roundness of the particles is written in gray squares below each sample.

The concentration of OA also influences the size of the nanoparticles. Figure 3 presents representative SEM images of particles from the three crystalline samples (0, 0.3, and 0.6 mol of OA). All particles exhibit a spherical morphology (refer to the Roundness definition in the experimental section) and show a trend in size corresponding to the OA concentration; as the OA concentration increases, the particle size decreases from ~30 nm to ~10 nm for the sample with 0 and 0.6 mol of OA, respectively (Figure 4). This trend is further supported by the diffractograms in Figure 1, where a peak broadening correlates with a reduction in crystallite size as described by the Scherrer equation. Additionally, the OA seems to act as a “homogenizer” regarding particle size distribution; increasing the OA concentration reduces the polydispersity and the presence of larger agglomerates, resulting in a more monodispersed particle distribution.

**Figure 5:**
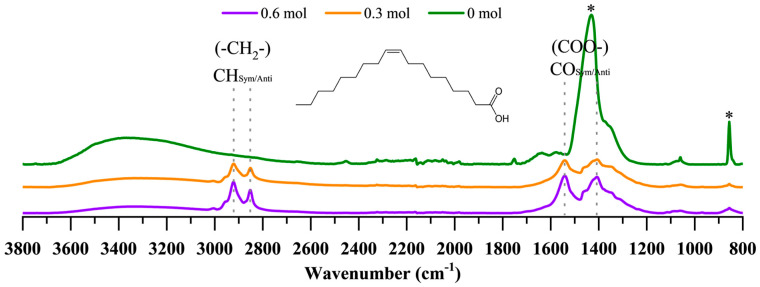
IR spectra of BT nanoparticles synthesized solvothermally at 200 °C for 3 h at three different OA concentrations. The representative bands for the deprotonated carboxylate group of the OA complexed with Ba/Ti are shown with dotted lines (CO_νsym_ = 1408 cm^−1^, CO_νasym_ = 1553 cm^−1^) as well as the bands corresponding to the aliphatic tail (CH_ν_ = 2853, 2924 cm^−1^), while for the BaCO_3_ bands are marked with an asterisk (*).

FTIR spectroscopy was employed to investigate the interactions between OA and BT nanoparticles (Figure 5). The sample synthesized without OA exhibited no characteristic vibrations (as expected), apart from those attributable to BaCO_3_ at ~1440 cm^−1^ and ~850 cm^−1^. In contrast, spectra from samples synthesized with OA displayed distinct vibrations corresponding to the deprotonated carboxylate group of OA at ~1408 cm^−1^ (symmetric stretching) and ~1553 cm^−1^ (asymmetric stretching), with a frequency difference (Δν ≈ 145 cm^−1^) indicative of a chelating bidentate binding mode [69,70]. These findings confirm effective surface bonding of OA to the nanoparticles. Based on these results, we propose that the chelating bidentate binding of OA to the BT surface is not random but instead promotes the formation of a relatively ordered organic shell around the inorganic core. This organized shell establishes a coherent interface with the BT lattice. The disparity in structure and lattice parameters between the crystalline BT core and the organic shell induces significant compressive strain at the interface. This surface-induced strain is identified as the primary factor influencing the phase stability of the nanoparticles, as substantiated by the W–H analysis (Table A2). Our proposed mechanism aligns with recent high-impact studies, such as the work of Tyson et al. (2023) [29], which demonstrated that BT nanoparticles synthesized via top-down ball-milling in the presence of oleic acid form a crystalline barium oleate shell in situ. This shell enhances and restores the ferroelectric (tetragonal) properties of the nanoparticles, an effect attributed directly to interfacial strain caused by lattice mismatch. Furthermore, the theoretical framework developed by Morozovska et al. (2007) [71] highlights the critical role of intrinsic surface stress and confinement effects in determining phase stability in ferroic nanoparticles like BT, even inducing phase transitions not observed in bulk materials.

Moreover, the FTIR spectra show a broad, weak peak near 3400 cm^−1^, indicative of hydroxyl groups adsorbed on the particle surfaces, while the absence of a sharp peak around 3500 cm^−1^ [72,73] suggests that protons and hydroxyl ions are not incorporated into the oxygen sublattice; the presence of these species is known to negatively affect the crystallinity and tetragonality of BT nanoparticles, as detailed in the introduction [13,23,24,25,26,27,74]. Employing an organic solvent such as methanol during synthesis, rather than water, minimizes hydroxyl incorporation within the nanoparticle lattice and thereby helps maintain the tetragonal phase of BT. Furthermore, Stawski et al. (2012) [75] demonstrated that hydroxyl defects contribute to local structural disorder and principally suppress the cubic-to-tetragonal phase transition at room temperature in nanoparticles synthesized by wet-chemical methods. In addition, Ji et al. (2022) [43] confirm that the BT phase is primarily influenced by the hydroxylation process due to its interaction with the solvent.

**Figure 6:**
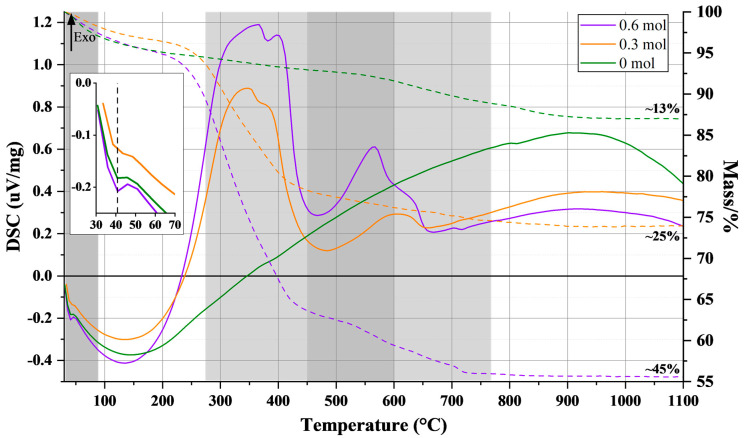
Representative TG–DTA curves of BT nanoparticles synthesized solvothermally at 200 °C for 3 h at three different OA concentrations; the DTA peak marked with a dotted line corresponds to the phase transition of BT from the tetragonal to the cubic phase.

Another significant consequence of the “size-effect” is the observed shift in the Curie transition temperature (Tc) between the cubic and tetragonal phases, which decreases from 120 °C in bulk BT [76] to lower values, approaching room temperature [4,45]. Figure 6 shows the TG–DTA curves for the three crystalline samples, with a detailed view around 50 °C. The DTA curves of all samples display an endothermic peak at ~40 °C, assigned to the tetragonal-cubic transition; this further supports the presence of tetragonal symmetry. This value for Tc has been previously reported for nanoparticles of comparable size [45,77,78].

Furthermore, the TG curves can be used to determine the extent of surface coverage by OA on the particles. The overall weight loss recorded between 30 °C and 1100 °C can be segmented into three parts for samples synthesized with OA. In the first segment, below 250 °C, all samples exhibit a 6% weight loss, attributed to the desorption of moisture and hydroxyl groups, which differ from lattice-incorporated hydroxyls [24] that do not appear in the FTIR spectra (as indicated by the absence of a sharp peak at approximately 3500 cm^−1^). Additionally, studies such as the work by Kholodkova et al. (2015) [79], using thermal analysis, have indicated that BT synthesized in water-containing fluids retains notable quantities of hydroxyl groups within its crystalline structure, necessitating high-temperature annealing (up to 1100 °C) for their complete removal.

The second segment, occurring below 450 °C, features exothermic peaks and weight losses of 1%, 16%, and 30%, corresponding to the decomposition of organic residues and fatty acids in samples synthesized with 0, 0.3, and 0.6 mol of OA, respectively. The subsequent exothermic peak around 550 °C in samples containing OA is associated with weight losses of 2% and 6% (for 0.3 and 0.6 mol of OA, respectively) and relates to larger molecules adsorbed on the nanoparticle’s surfaces. These molecules are byproducts from a side reaction of OA in methanol under basic conditions; previous studies indicate that the C=C double bond in the aliphatic tail may oxidize or polymerize [80,81,82]. This interpretation is supported by FTIR spectra in Figure 5, which show no C=C stretching vibration near 1600 cm^−1^, indicating transformation of the double bond. The final stage, above 600 °C, involves the decomposition of residual BaCO_3_, with weight losses of 6%, 1%, and 3% for samples synthesized with 0, 0.3, and 0.6 mol of OA, respectively.

### 3.2. Solvothermal Conditions

**Figure 7:**
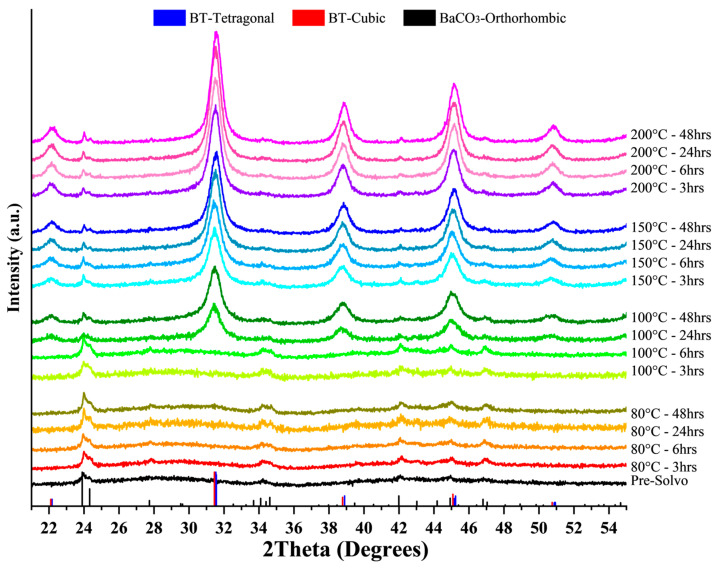
Diffractograms of BT nanoparticles synthesized under different solvothermal conditions and OA concentrations of 0.3 mol (BT:OA)/(1:0.3). As a reference, the relative peak intensities for the tetragonal (PDF No. 98-005-7485) and cubic (PDF No. 98-008-0552) phase of BT are shown, as well as for BaCO_3_ (PDF No. 01-074-2663).

**Figure A1:**
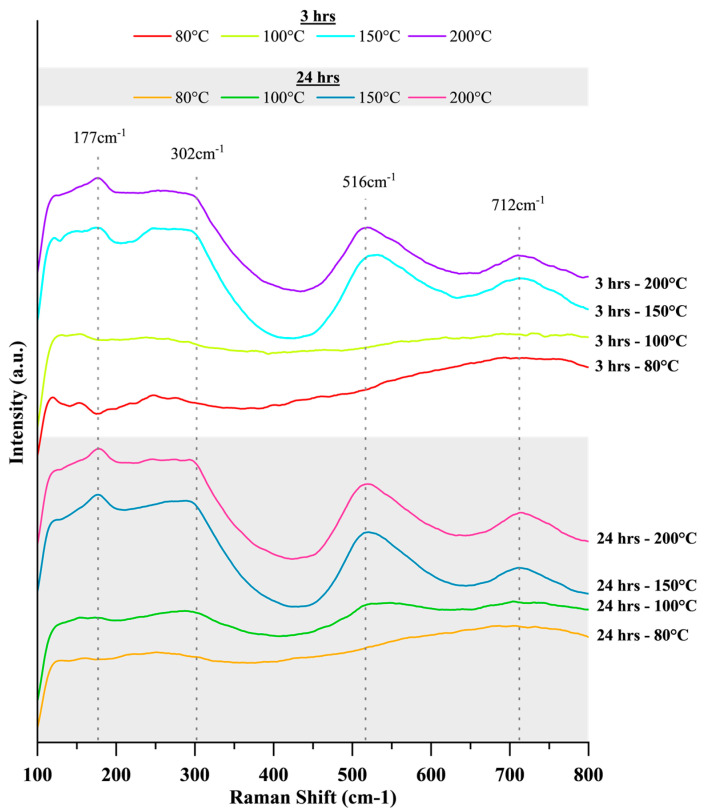
Representative Raman spectra of BT nanoparticles synthesized under different solvothermal conditions (temperature and length). The representative bands for tetragonal BT are shown with dotted lines.

**Figure A2:**
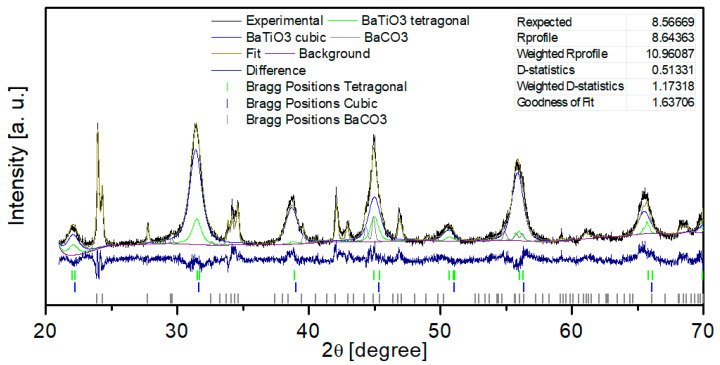
Rietveld refinement of the diffractogram of BT nanoparticles synthesized solvothermally at 200 °C for 3 h without OA. As a reference, the relative peak intensities for the tetragonal (PDF No. 98-005-7485) and cubic (PDF No. 98-008-0552) phases of BT are shown, as well as for BaCO_3_ (PDF No. 01-074-2663). The crystallographic data used for the fitting process was as follows: BaTiO_3_ (cubic): ICSD 98-002-8848. BaTiO_3_ (tetragonal): ICSD 98-02-8620. BaCO_3_ (orthorhombic): ICSD 98-001-5196.

**Figure A3:**
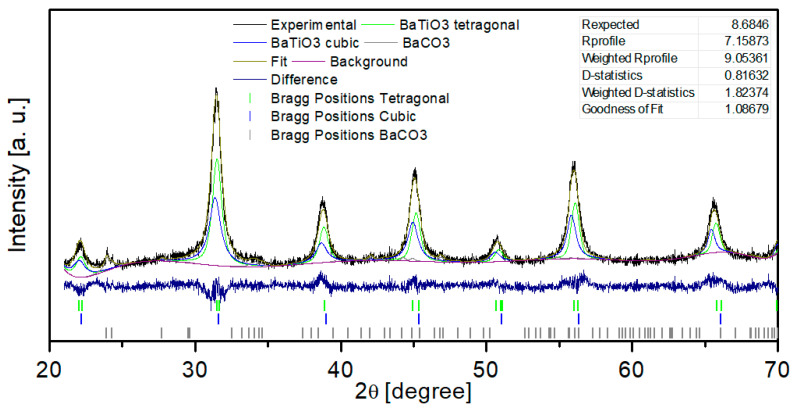
Rietveld refinement of the diffractogram of BT nanoparticles synthesized solvothermally at 200 °C for 3 h with 0.3 mol of OA. As a reference, the relative peak intensities for the tetragonal (PDF No. 98-005-7485) and cubic (PDF No. 98-008-0552) phases of BT are shown, as well as for BaCO_3_ (PDF No. 01-074-2663). The crystallographic data used for the fitting process was as follows: BaTiO_3_ (cubic): ICSD 98-002-8848. BaTiO_3_ (tetragonal): ICSD 98-02-8620. BaCO_3_ (orthorhombic): ICSD 98-001-5196.

**Figure A4:**
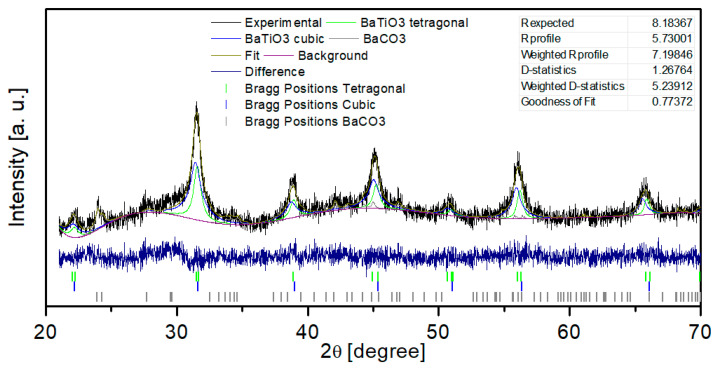
Rietveld refinement of the diffractogram of BT nanoparticles synthesized solvothermally at 200 °C for 3 h with 0.6 mol of OA. As a reference, the relative peak intensities for the tetragonal (PDF No. 98-005-7485) and cubic (PDF No. 98-008-0552) phases of BT are shown, as well as for BaCO_3_ (PDF No. 01-074-2663). The crystallographic data used for the fitting process was as follows: BaTiO_3_ (cubic): ICSD 98-002-8848. BaTiO_3_ (tetragonal): ICSD 98-02-8620. BaCO_3_ (orthorhombic): ICSD 98-001-5196.

Building upon the previously described synthesis route, which effectively yielded OA-capped BT nanoparticles, the influence of the solvothermal conditions—specifically temperature and duration—was systematically investigated. An OA concentration of 0.3 mol (BT:OA)/(1:0.3) was selected for these experiments due to its association with the highest fraction of tetragonal BT according to the Rietveld refinement. Figure 7 presents the diffractograms of samples synthesized under different solvothermal conditions, while Figure A1 presents the Raman spectra of selected samples. The diffractograms show a progression from amorphous to crystalline phases as both temperature and reaction time increase; minor amounts of BaCO_3_ are detected in all samples. Crystalline samples exhibit a mixed cubic/tetragonal phase, whereas crystalline BT could not be achieved at 80 °C even after 48 h of synthesis. Nevertheless, amorphous samples displayed weak Raman features—presumably characteristic of tetragonal BT—even for those synthesized at 80 °C for 3 h. This observation may be attributed to the presence of incipient BT crystallites. As documented in prior studies [18,61,83,84], BT synthesis via a sol–gel-precipitation method comprises two principal stages: rapid formation of titanium-gel nanoparticles, followed by gradual precipitation and crystallization of BT. During the early portion of the second stage, barium ions diffuse into the amorphous titanium gel and adsorb onto its surface, initiating crystallization and leading to the formation of nanoscale crystallites that may explain the observed Raman signals despite their absence in XRD.

First, if the OA is cross-linked and packed as multi-layers at the surface of the particles, then the OA would not only hinder the growth of the particles by slowing the diffusion, but also by mechanically restricting it as a cage. Second, if the growth of the nanoparticles proceeds by aggregation, where the hydroxyl groups at the surface of the particles condense to form new M-O-M bonds [85], then the unavailability of the hydroxyl groups due to the capped OA would prevent the growth. Indeed, the cross-linking of OA and the existence of multi-layers are supported by the IR spectra (Figure 5) and the TG–DTA curves (Figure 6).

**Figure 8:**
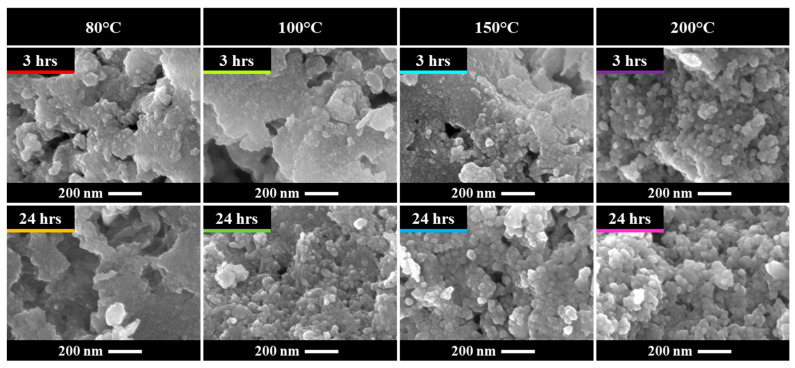
Representative SEM images of BT nanoparticles synthesized under different solvothermal conditions and OA concentrations of 0.3 mol (BT:OA)/(1:0.3).

**Figure 9:**
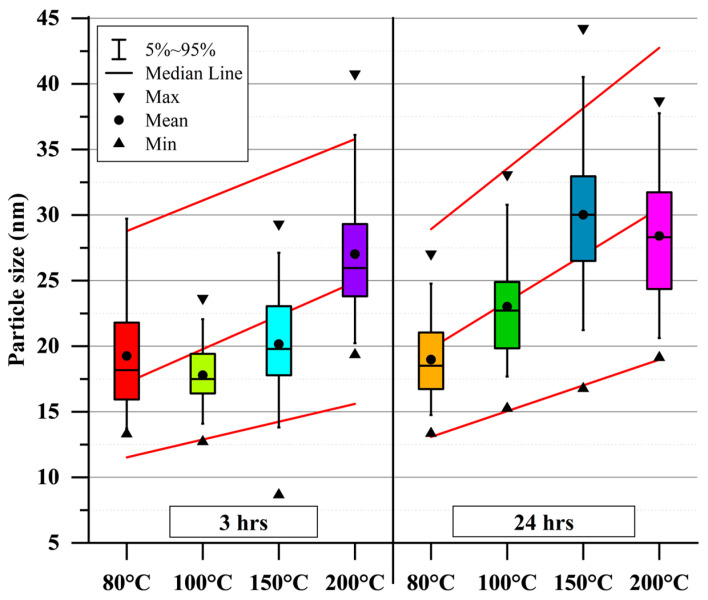
Particle size (from SEM images) of BT nanoparticles synthesized under different solvothermal conditions and an OA concentration of 0.3 mol; the maximum, minimum, and mean particle sizes were fitted (dotted lines) as a reference for the reader.

**Figure 10:**
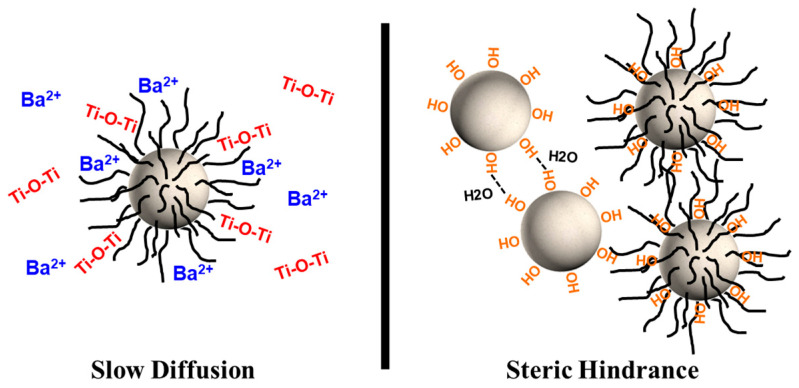
Representation of the two ways OA may hinder the growth of the particles: diffusion and steric.

The influence of solvothermal conditions on particle size is illustrated in Figure 8 and Figure 9. All particles exhibit a spherical shape (with roundness values between 1.06 and 1.13) and have an average particle size ranging from ~15 nm to ~30 nm, with size increasing as temperature rises. For samples synthesized at 100 °C and 150 °C, the mean particle size increases over time, whereas at 80 °C and 200 °C, no significant change in mean particle size is observed. The growth limitation of particles to approximately ~30 nm may be attributed to two factors (Figure 10). Firstly, if OA forms layers on the particle’s surface, the OA could hinder particle growth both by slowing diffusion and imposing mechanical restrictions. Secondly, if nanoparticle growth occurs through aggregation facilitated by condensation of surface hydroxyl groups to form new M–O–M bonds [85], then capping of hydroxyl groups by OA would inhibit further growth. The last is supported by the FTIR spectra (Figure 5) and TG–DTA curves (Figure 6).

## 4. Conclusions

This study presents, for the first time, a one-pot, water-free solvothermal approach using a Ti(O-iPr)_4_-Ba(OH)_2_·8H_2_O sol in methanol, incorporating small amounts of OA and relying solely on the hydration water from the Ba(OH)_2_·8H_2_O precursor as a controlled water source. Crystalline BT nanoparticles were synthesized at moderate temperatures (as low as 100 °C) without the necessity for post-synthetic calcination. XRD analysis confirmed a cubic/tetragonal crystal structure, with significant tetragonal content indicated by Raman and Rietveld refinement. The resulting particles are homogeneous in morphology and size with almost no agglomeration and possess an apparent spherical shape with diameters ranging from ~12 nm to ~30 nm, depending on synthesis conditions. Notably, the retention of the tetragonal phase at these small sizes is attributed to the absence of lattice hydroxyls—verified by FTIR spectroscopy—due to the use of methanol as the organic solvent, in contrast to traditional hydrothermal or water-based methods. Additionally, small quantities of OA proved to favor the tetragonal phase, while larger amounts promoted the cubic phase, likely due to greater compressive stress on the particles induced by the OA. This synthesis strategy is particularly advantageous because: (1) lower reaction temperatures facilitate the production of discrete nanoscale particles and enhance economic feasibility; (2) the use of only barium hydroxide and titanium(IV) isopropoxide results in powders free from inorganic impurities such as alkali metals or halides, thus minimizing the need for extensive washing; (3) all starting materials are commercially available; and (4) crystalline BT is obtained directly.

## Data Availability

The dataset is available on request from the authors.

## Appendix A

**Table A1:** Summary of the phase composition/fraction obtained by Rietveld refinement of the diffractograms of BT nanoparticles synthesized solvothermally at 200 °C for 3 h at 3 different OA concentrations.

BT:OA	Tetragonal	Cubic	BaCO_3_
1:0	15.1%	63.1%	21.8%
1:0.3	56.0%	39.6%	4.4%
1:0.6	36.9%	53.9%	9.2%

**Table A2:** Summary of the calculated crystallite size and lattice strain values obtained by the Williamson–Hall method, as well as crystallite size calculated by the Scherrer equation.

	Williamson-Hall		Scherrer Diameter (nm)	
	Diameter (nm)	Strain	(101)/(110)	Average
BaTiO_3_ tetragonal 0.0	5.84	0.00737	8.66	11.80
BaTiO_3_ cubic 0.0	5.31	0.00475	6.51	7.38
BaTiO_3_ tetragonal 0.3	6.64	0.00532	9.24	11.10
BaTiO_3_ ubic 0.3	4.66	0.00842	6.67	8.58
BaTiO_3_ tetragonal 0.6	6.98	0.00554	9.93	12.59
BaTiO_3_ cubic 0.6	3.75	0.00998	5.34	6.72

## References

[1] (2014). Market Report—Global Piezoelectric Device Market.

[2] Arlt G., Hennings D., de With G. (1985). Dielectric properties of fine grained barium titanate ceramics. J. Appl. Phys..

[3] Uchino K., Sadanaga E., Hirose T. (1989). Dependence of the crystal structure on particle size in barium titanate. J. Am. Ceram. Soc..

[4] Frey M., Payne D. (1996). Grain-size effect on structure and phase transformations for barium titanate. Phys. Rev. B Condens. Matter.

[5] Spanier J.E., Kolpak A.M., Urban J.J., Grinberg I., Ouyang L., Yun W.S., Rappe A.M., Park H. (2006). Ferroelectric phase transition in individual single-crystalline BaTiO_3_ nanowires. Nano Lett..

[6] Hoshina T., Wada S., Kuroiwa Y., Tsurumi T. (2008). Composite structure and size effect of barium titanate nanoparticles. Appl. Phys. Lett..

[7] Hoshina T. (2013). Size effect of barium titanate: Fine particles and ceramics. J. Ceram. Soc. Jpn..

[8] Jaccard C., Kaenzig W., Peter M. (1953). Das Verhalten von kolloidalen Seignetteelektrika. I, Kaliumphosphat KH_2_PO_4_. Helv. Phys. Acta.

[9] Zhu J., Han W., Zhang H., Yuan Z., Wang X., Li L., Jin C. (2012). Phase coexistence evolution of nano BaTiO_3_ as function of particle sizes and temperatures. J. Appl. Phys..

[10] Mueller V., Beige H., Abicht H.-P., Eisenschmidt C. (2011). X-ray diffraction study revealing phase coexistence in barium titanate stannate. J. Mater. Res..

[11] Jacobs A. (2000). Landau theory of structures in tetragonal-orthorhombic ferroelastics. Phys. Rev. B.

[12] Polking M.J., Alivisatos P., Ramesh R. (2015). Synthesis, physics, and applications of ferroelectric nanomaterials. MRS Commun..

[13] Yoon D.H. (2006). Tetragonality of barium titanate powder for a ceramic capacitor application. J. Ceram. Process. Res..

[14] Bell A.J. Grain size effects in barium titanate-revisited. Proceedings of the 1994 IEEE International Symposium on Applications of Ferroelectrics.

[15] Pandey D., Singh P., Tiwari V.S. (1992). Developments in ferroelectric ceramics for capacitor applications. Bull. Mater. Sci..

[16] Tan Y., Zhang J., Wu Y., Wang C., Koval V., Shi B., Ye H., McKinnon R., Viola G., Yan H. (2015). Unfolding grain size effects in barium titanate ferroelectric ceramics. Sci. Rep..

[17] Lines M.E., Glass A.M. (1977). Principles and Applications of Ferroelectrics and Related Materials.

[18] Pithan C., Hennings D., Waser R. (2005). Progress in the synthesis of nanocrystalline BaTiO_3_ powders for MLCC. Int. J. Ceram. Technol..

[19] Simon-Seveyrat L., Hajjaji A., Emziane Y., Guiffard B., Guyomar D. (2007). Re-investigation of synthesis of BaTiO_3_ by conventional solid-state reaction and oxalate coprecipitation route for piezoelectric applications. Ceram. Int..

[20] Zhu X., Zhu J., Zhou S., Liu Z., Ming N., Hesse D. (2005). BaTiO_3_ nanocrystals: Hydrothermal synthesis and structural characterization. J. Cryst. Growth.

[21] Makino T., Arimura M., Fujiyoshi K., Yamashita Y., Kuwabara M. (2007). Crystallinity of Barium Titanate Nanoparticles Synthesized by Sol-Gel Method. Key Eng. Mater..

[22] Gomes M.A., Lima Á.S., Eguiluz K.I.B., Salazar-Banda G.R. (2016). Wet chemical synthesis of rare earth-doped barium titanate nanoparticles. J. Mater. Sci..

[23] Sasirekha N., Rajesh B. (2008). Hydrothermal Synthesis of Barium Titanate: Effect of Titania Precursor and Calcination Temperature on Phase Transition. Ind. Eng. Chem. Res..

[24] Hennings D., Schreinemacher S. (1992). Characterization of hydrothermal barium titanate. J. Eur. Ceram. Soc..

[25] Vivekanandan R., Kutty T.R.N. (1989). Characterization of barium titanate fine powders formed from hydrothermal crystallization. Powder Technol..

[26] Busca G., Buscaglia V., Leoni M., Nanni P. (1994). Solid-State and Surface Spectroscopic Characterization of BaTiO_3_ Fine Powders. Chem. Mater..

[27] Hennings D.F.K., Metzmacher C., Schreinemacher B.S. (2001). Defect Chemistry and Microstructure of Hydrothermal Barium Titanate. J. Am. Ceram. Soc..

[28] Clark I., Sinclair D. (1999). Hydrothermal synthesis and characterisation of BaTiO_3_ fine powders: Precursors, polymorphism and properties. J. Mater. Chem..

[29] Zhang H., Liu S., Ghose S., Ravel B., Idehenre I.U., Barnakov Y.A., Basun S.A., Evans D.R., Tyson T.A. (2023). Structural origin of recovered ferroelectricity in BaTiO_3_ nanoparticles. Phys. Rev. B.

[30] Philippot G., Elissalde C., Maglione M., Aymonier C. (2014). Supercritical fluid technology: A reliable process for high quality BaTiO_3_ based nanomaterials. Adv. Powder Technol..

[31] Niederberger M., Pinna N. (2009). Metal Oxide Nanoparticles in Organic Solvents.

[32] Vioux A. (1997). Nonhydrolytic Sol−Gel Routes to Oxides. Chem. Mater..

[33] Niederberger M. (2007). Nonaqueous sol-gel routes to metal oxide nanoparticles. Acc. Chem. Res..

[34] Garnweitner G., Hentschel J., Antonietti M., Niederberger M. (2005). Nonaqueous Synthesis of Amorphous Powder Precursors for Nanocrystalline PbTiO_3_, Pb(Zr,Ti)O_3_, and PbZrO_3_. Chem. Mater..

[35] Dosch R. (1984). Preparation of barium titanate films using sol-gel techniques. MRS Proc..

[36] Takashi H., Kimihiko S., Katsuaki S. (1995). Chemical processing and dielectric properties of BaTiO_3_ ceramics. Ceram. Trans..

[37] Niederberger M., Pinna N., Polleux J., Antonietti M. (2004). A general soft-chemistry route to perovskites and related materials: Synthesis of BaTiO_3_, BaZrO_3_, and LiNbO_3_ nanoparticles. Angew. Chem. Int. Ed. Engl..

[38] Djerdj I., Arčon D., Jagličić Z., Niederberger M. (2008). Nonaqueous synthesis of metal oxide nanoparticles: Short review and doped titanium dioxide as case study for the preparation of transition metal-doped oxide nanoparticles. J. Solid. State Chem..

[39] Santos G.O.S., Silva R.S., Costa L.P., Cellet T.S.P., Rubira A.F., Eguiluz K.I.B., Salazar-Banda G.R. (2014). Influence of synthesis conditions on the properties of electrochemically synthesized BaTiO_3_ nanoparticles. Ceram. Int..

[40] Zhang M., Caldwell T., Hector A.L., Garcia-Araez N., Falvey J. (2023). Solvothermal synthesis of nanoscale BaTiO_3_ in benzyl alcohol–water mixtures and effects of manganese oxide coating to enhance the PTCR effect. Dalton Trans..

[41] Taleb S., Badillo M., Flores-Ruiz F.J., Acuautla M. (2023). From synthesis to application: High-quality flexible piezoelectric sensors fabricated from tetragonal BaTiO_3_/P(VDF-TrFE) composites. Sens. Actuators A Phys..

[42] Zhang Y.C., Wang G.L., Li K.W., Zhang M., Hu X.Y., Wang H. (2006). Facile synthesis of submicron BaTiO_3_ crystallites by a liquid–solid reaction method. J. Cryst. Growth.

[43] Ji X., Zhu Y., Lian X., Fan B., Liu X., Xiao P., Zhang Y. (2022). Hydroxylation mechanism of phase regulation of nanocrystal BaTiO_3_ synthesized by a hydrothermal method. Ceram. Int..

[44] Zhang Q., Jia Y., Wu W., Pei C., Zhu G., Wu Z., Zhang L., Fan W., Wu Z. (2023). Review on strategies toward efficient piezocatalysis of BaTiO_3_ nanomaterials for wastewater treatment through harvesting vibration energy. Nano Energy.

[45] Mao Y., Mao S., Ye G., Xie Z., Zheng L. (2010). Solvothermal synthesis and Curie temperature of monodispersed barium titanate nanoparticles. Mater. Chem. Phys..

[46] Cho C.-R., Lee S.-J., Jang M.-S., Kim H.-J., Jeong S., Ro D.-T., Kim S.-C. (1992). Ferroelectric BaTiO_3_ thin films and ceramics fabrication by sol-gel synthesis. Korean Phys. Soc..

[47] Hayashi T., Shinozaki H., Sasaki K. (1999). Preparation and properties of (Ba0·7Sr0·3)TiO_3_ powders and thin films using precursor solutions formed from alkoxide-hydroxide. J. Eur. Ceram. Soc..

[48] Upadhyah R.H., Argekar A.P., Deshmukh R.R. (2014). Characterization, dielectric and electrical behaviour of BaTiO_3_ nanoparticles prepared via Titanium (IV) triethanolaminato isopropoxide and hydrated Barium. Bull. Mater. Sci..

[49] Chen D., Jiao X. (2004). Solvothermal Synthesis and Characterization of Barium Titanate Powders. J. Am. Ceram. Soc..

[50] O’Brien S., Brus L., Murray C.B. (2001). Synthesis of Monodisperse Nanoparticles of Barium Titanate: Toward a Generalized Strategy of Oxide Nanoparticle Synthesis. J. Am. Chem. Soc..

[51] Niederberger M., Garnweitner G. (2005). Nonaqueous synthesis of barium titanate nanocrystals in acetophenone as oxygen supplying agent. MRS Proc..

[52] Smith M.B., Page K., Siegrist T., Redmond P.L., Walter E.C., Seshadri R., Brus L.E., Steigerwald M.L. (2008). Crystal structure and the paraelectric-to-ferroelectric phase transition of nanoscale BaTiO_3_. J. Am. Chem. Soc..

[53] Kwon S.-G., Choi K., Kim B.-I. (2006). Solvothermal synthesis of nano-sized tetragonal barium titanate powders. Mater. Lett..

[54] Kwon S., Park B., Choi K., Choi E., Nam S., Kim J. (2006). Solvothermally synthesized tetragonal barium titanate powders using H_2_O/EtOH solvent. J. Eur. Ceram. Soc..

[55] Wei X., Xu G., Ren Z., Wang Y., Shen G., Han G. (2007). Synthesis of Highly Dispersed Barium Titanate Nanoparticles by a Novel Solvothermal Method. J. Am. Ceram. Soc..

[56] Ohno T., Suzuki D., Suzuki H., Ida T. (2004). Size effect for barium titanate nano-particles. Kona.

[57] Robins L., Kaiser D., Rotter L., Schenck P. (1994). Investigation of the structure of barium titanate thin films by Raman spectroscopy. J. Appl..

[58] DiDomenico M., Wemple S.H., Porto S.P.S., Bauman R.P. (1968). Raman Spectrum of Single-Domain BaTiO_3_. Phys. Rev..

[59] El Marssi M., Le Marrec F., Lukyanchuk I.A., Karkut M.G. (2003). Ferroelectric transition in an epitaxial barium titanate thin film: Raman spectroscopy and X-ray diffraction study. J. Appl. Phys..

[60] Fang C., Zhou D., Gong S. (2011). Core-shell structure and size effect in barium titanate nanoparticle. Phys. B Condens. Matter.

[61] Rabuffetti F.A., Brutchey R.L. (2012). Structural evolution of BaTiO_3_ nanocrystals synthesized at room temperature. J. Am. Chem. Soc..

[62] Bakhtbidar M., Merlen A., Ruediger A. (2025). Ferroelectric-to-paraelectric phase transition probing via high-resolution tip-enhanced Raman spectroscopy. Opt. Commun..

[63] Surmenev R.A., Chernozem R.V., Skirtach A.G., Bekareva A.S., Leonova L.A., Mathur S., Ivanov Y.F., Surmeneva M.A. (2021). Hydrothermal synthesis of barium titanate nano/microrods and particle agglomerates using a sodium titanate precursor. Ceram. Int..

[64] Duong N.X., Bae J.S., Jeon J., Lim S.Y., Oh S.H., Ullah A., Sheeraz M., Choi J.S., Ko J.H., Yang S.M. (2019). Polymorphic phase transition in BaTiO_3_ by Ni doping. Ceram. Int..

[65] Lin S., Lü T., Jin C., Wang X. (2006). Size effect on the dielectric properties of BaTiO_3_ nanoceramics in a modified Ginsburg-Landau-Devonshire thermodynamic theory. Phys. Rev. B.

[66] Arlt G. (1990). The influence of microstructure on the properties of ferroelectric ceramics. Ferroelectrics.

[67] Murugesan C., Chandrasekaran G. (2015). Impact of Gd3+ substitution on the structural, magnetic and electrical properties of cobalt ferrite nanoparticles. RSC Adv..

[68] Ahlawat A., Sathe V.G., Reddy V.R., Gupta A. (2011). Mossbauer, Raman and X-ray diffraction studies of superparamagnetic NiFe_2_O_4_ nanoparticles prepared by sol–gel auto-combustion method. J. Magn. Magn. Mater..

[69] Nara M., Torii H., Tasumi M. (1996). Correlation between the vibrational frequencies of the carboxylate group and the types of its coordination to a metal ion: An ab initio molecular orbital study. J. Phys. Chem..

[70] Cai Q.J., Gan Y., Chan-Park M.B., Yang H.B., Lu Z.S., Li C.M., Guo J., Dong Z.L. (2009). Solution-Processable Barium Titanate and Strontium Titanate Nanoparticle Dielectrics for Low-Voltage Organic Thin-Film Transistors. Chem. Mater..

[71] Morozovska A.N., Glinchuk M.D., Eliseev E.A. (2007). Phase transitions induced by confinement of ferroic nanoparticles. Phys. Rev. B Condens. Matter Mater. Phys..

[72] Gomi K., Tanaka K., Kamiya H. (2004). Effect of Mixing Condition on Sol-Gel Synthesis of Barium Titanate Ultrafine Particles [Translated]†. KONA Powder Part. J..

[73] Wada S., Suzuki T., Noma T. (1996). Role of Lattice Defects in the Size Effect of Barium Titanate Fine Particles. J. Ceram. Soc. Jpn..

[74] Yoon S., Baik S., Kim M.G., Shin N. (2006). Formation Mechanisms of Tetragonal Barium Titanate Nanoparticles in Alkoxide-Hydroxide Sol-Precipitation Synthesis. J. Am. Ceram. Soc..

[75] Stawski T.M., Veldhuis S.A., Göbel O.F., Podstawka-Proniewicz E., Elshof J.E.T. (2012). Electron microscopy study of intragranular nanoporosity and the occurrence of local structural disorder in cubic BaTiO_3_ nanopowders from alkoxide–hydroxide precipitation process. Ceram. Int..

[76] Wakaki M., Shibuya T., Kudo K. (2007). Physical Properties and Data of Optical Materials.

[77] Ma Q., Mimura K., Kato K. (2014). Diversity in size of barium titanate nanocubes synthesized by a hydrothermal method using an aqueous Ti compound. CrystEngComm.

[78] Szwarcman D., Vestler D., Markovich G. (2011). The size-dependent ferroelectric phase transition in BaTiO_3_ nanocrystals probed by surface plasmons. ACS Nano.

[79] Kholodkova A.A., Danchevskaya M.N., Ivakin Y.D., Muravieva G.P. (2015). Synthesis of fine-crystalline tetragonal barium titanate in low-density water fluid. J. Supercrit. Fluids.

[80] Xia Y., Larock R.C. (2010). Vegetable oil-based polymeric materials: Synthesis, properties, and applications. Green Chem..

[81] Shahidi F. (2005). Bailey’s Industrial Oil and Fat Products.

[82] Renz M. (2005). Ketonization of Carboxylic Acids by Decarboxylation: Mechanism and Scope. Eur. J. Org. Chem..

[83] Hennings D., Rosenstein G., Schreinemacher H. (1991). Hydrothermal preparation of barium titanate from barium-titanium acetate gel precursors. J. Eur. Ceram. Soc..

[84] Kageyama H., Oaki Y., Takezawa Y. (2013). Low-temperature syntheses of cubic BaTiO_3_ nanoparticles in highly basic aqueous solution. J. Ceram. Soc. Jpn..

[85] Simonsen M.E., Søgaard E.G. (2009). Sol–gel reactions of titanium alkoxides and water: Influence of pH and alkoxy group on cluster formation and properties of the resulting products. J. Solgel Sci. Technol..

